# Editorial: Impact of inhaled particulates and toxicants on brain health: inflammation mechanisms and behavioral consequences

**DOI:** 10.3389/fnbeh.2026.1937586

**Published:** 2026-09-14

**Authors:** Racheal O. Asaolu, Jagan Srinivasan, Benjamin C. Nephew

**Affiliations:** Worcester Polytechnic Institute, Worcester, MA, United States

**Keywords:** behavior, brain health, inflammation, inhaled particulates, toxicants

Air pollution has long been recognized as a major threat to respiratory and cardiovascular health, yet growing evidence indicates that its effects extend well-beyond these systems to the brain and neurodegenerative diseases. As urbanization, industrialization, and climate change continue to reshape environmental exposures, inhaled particulate matter (PM) and associated toxicants have emerged as important contributors to neurological dysfunction and neurodegenerative disease. The increasing prevalence of Alzheimer's disease, Parkinson's disease, and related disorders has intensified interest in understanding how environmental factors influence brain health. Evidence from epidemiological, toxicological, and experimental studies suggest that inhaled particulates are not merely respiratory irritants but systemic toxicants capable of disrupting brain function through complex and interconnected biological mechanisms. This Research Topic brings together multidisciplinary perspectives that advance our understanding of how inhaled particulates and toxicants initiate oxidative stress, inflammatory responses, disrupt neural homeostasis, and alter behavior across experimental systems.

Collectively, the studies reflect the evolution of environmental neuroscience from identifying associations between pollution and disease toward defining the biological mechanisms responsible for neurological injury. Rather than acting through a single pathway, inhaled particles initiate interconnected processes involving oxidative stress, neuroinflammation, mitochondrial dysfunction, vascular injury, impaired protein homeostasis, and blood-brain barrier disruption. Together, these studies demonstrate that environmental toxicants are systemic stressors capable of influencing neurological health across the lifespan.

Song et al. present the development of the Anhui Environmental Exposure Questionnaire (AEEQ) to integrate chronic ambient/indoor pollution and sleep-quality metrics, two key inter-related factors for neurodegenerative disorders and associated cognitive impairment. This novel survey has robust psychometric performance and independently predicted 24-month risk of cerebral hemorrhage in community-dwelling older adults. It impressively provides graded exposure response and incremental discrimination that can significantly augment current risk stratification and enhance targeted and feasible prevention given there are effective methods for both decreasing pollution exposure and treating sleep disorders. While further validation is needed, the practical application potential of the AEEQ to prevent pollution and sleep related cognitive impairment in the near future is substantial.

One major general challenge in studies of particulate matter exposures and brain health is exposure specificity. Given that it is established that aluminum exposure, often through particulates, is linked to cognitive impairment, Wang et al. focused on relationships between plasma aluminum concentration, hemoglobin-to-red cell distribution width ratio (HRR), a key inflammatory marker, and cognitive function in high risk aluminum workers. They report increased risk of cognitive impairment with increased plasma aluminum concentrations, which was partially explained by decreased HRR. This work could lead to improved exposure monitoring, worker safety, and prevention of aluminum associated cognitive impairment in aluminum workers.

In a similar vein, Cory-Slechta et al. investigated the effects of inhaled iron or iron combined with sulfur dioxide in a naturalistic mouse model which included developmental and subsequent later life behavioral exposure to address the reality of multiple exposures at different life history stages. The behavioral exposure unmasked sex-specific effects (another topic that is often overlooked) on neurochemical factors which may be relevant to air pollution associated neurodegeneration. This type of targeted, yet also naturalistic, work can inform regulatory decision making related to long term risks of air pollution exposure to the brain and cognition.

Finally, Marin-Castaneda et al. address the mixed literature on ozone exposure and neurodegenerative disorders with a timely brief review of recent studies. They underscore novel findings indicating that peripheral biomarkers may mediate the adverse effects of ozone on the brain and cognitive function, with a focus on the role of oxidative stress and immune dysregulation in Alzheimer's disease progression. The authors bring attention to bidirectional functions of the lung-brain axis and the general importance of peripheral processes in the pathological impacts of ozone in the central nervous system. Similar to increasing awareness of the risk of iron, and aluminum exposure, this review further highlights the importance of considering environmental health management in efforts to address high and increasing rates of neurodegenerative disorders and dementia.

An important message emerging from the articles is that particulate matter should not be regarded as a single pollutant. Fine and ultrafine particles differ substantially in size, chemistry, surface reactivity, and associated contaminants, all of which influence their biological effects. Future work should therefore move beyond measuring PM mass alone toward identifying the components and exposure scenarios that present the greatest neurological risk.

Ultimately, the studies assembled in this topic reinforce a fundamental shift in environmental health research: inhaled particulates and toxicants should no longer be viewed solely as respiratory hazards but as important determinants of brain health. By integrating mechanistic toxicology, exposure science, experimental neuroscience, and translational research, the Research Topic provides compelling evidence that air pollution contributes to neurological dysfunction through interconnected biological pathways. Continued interdisciplinary collaboration among toxicologists, neuroscientists, epidemiologists, clinicians, environmental scientists, and policy makers will be essential for translating these discoveries into effective public health policies, therapeutic strategies, and interventions that reduce the neurological burden of environmental pollution. Collectively, these contributions establish a strong foundation for future research while emphasizing the importance of protecting brain health from the adverse effects of inhaled particulates and toxicants.

